# Particulate matter exposure is highly correlated to pediatric asthma exacerbation

**DOI:** 10.18632/aging.203281

**Published:** 2021-07-13

**Authors:** Xin Yang, Yuanyuan Zhang, Xueqin Zhan, Xuchen Xu, Shuxian Li, Xuefeng Xu, Songmin Ying, Zhimin Chen

**Affiliations:** 1 Department of Pulmonology, Children's Hospital, Zhejiang University School of Medicine, National Clinical Research Center for Child Health, National Children's Regional Medical Center, Hangzhou 310052, China; 2 Department of Rheumatology Immunology and Allergy, Children's Hospital, Zhejiang University School of Medicine, National Clinical Research Center for Child Health, National Children's Regional Medical Center, Hangzhou 310052, China; 3 Department of Pharmacology and Department of Respiratory and Critical Care Medicine of the Second Affiliated Hospital, Zhejiang University School of Medicine, Key Laboratory of Respiratory Disease of Zhejiang Province, Hangzhou 310009, China; 4 International Institutes of Medicine, The Fourth Affiliated Hospital of Zhejiang University School of Medicine, Yiwu 322000, China

**Keywords:** asthma, airway inflammation, emergency department visits, particulate matter, pediatrics

## Abstract

Asthma is a heterogeneous disease in which environmental factors play an important role, and the effect of particulate matter (PM) on the occurrence and severity of asthma is drawing more attention. This study aims to identify the correlation between PM and pediatric asthma exacerbation and explore the potential mechanisms. The asthma visits data (N = 16,779,739) in a university-based tertiary children’s hospital from January 2013 to December 2017 were collected, and the relationship between asthma visits and local PM concentration was analyzed. For further study, we established a house dust mite (HDM)-induced allergic airway inflammation model with PM intervention. We detected a correlation between PM concentration and pediatric asthma visits, especially in children under 6 years old. The *in vivo* data showed that PM aggravated HDM-induced airway inflammation, and IL-33 neutralizing antibody exerted a protective role. Our study suggests that PM is a risk factor in promoting pediatric asthma exacerbation, in which IL-33 might be a promising target.

## INTRODUCTION

Asthma is one of the most common chronic diseases in children and adults. As the main cause of emergency department visits and hospitalizations, asthma severely affects patients’ quality of life [1]. Over the past few decades, the prevalence of asthma has continuously increased, which may be mainly due to environmental pollution caused by industrialization and urbanization [2, 3]. It has been confirmed that unfavorable meteorological factors, including high temperature, increased concentrations of greenhouse gases, and fine particulate pollution, affect patients with existing respiratory diseases and increase the incidence and prevalence of respiratory diseases and epidemic trends [4–6].

Among meteorological factors, air pollution is the most closely related to respiratory health. Particulate matter (PM) is one of the main components of air pollutants, which is mainly composed of coal combustion, automobile exhaust, dust from construction sites, and smoke from combustions [7]. Because of its physicochemical characteristics, PM can adsorb harmful substances on the surface and persist in the air for a long time [8]. With the process of gas exchange, PM enters the peripheral respiratory system or even alveoli and causes direct cell damage, which leads to multiple diseases, such as asthma, tumors, and acute coronary events [9, 10].

Previous epidemiological studies have reported that PM likely aggravates the respiratory symptoms and increases the morbidity and emergency department visits of asthma patients [11, 12]. It has been confirmed that multiple prolonged exposures to PM can exacerbate airway inflammation in the house dust mite (HDM)-induced asthma model and the pathological mechanisms might be related to the transforming growth factor-beta (TGF-β), PD-L1, TH17 cells, and Treg cells [13–15].

Due to the differences in the natural environment and economic status in regions, air pollution and disease occurrence also differ between regions. Moreover, few studies have focused on the effects of short-term PM exposure on airway inflammation in asthma. Therefore, it is urgent to understand the relationship between PM concentration and asthma in a particular region and the impact on airway inflammation posed by short-term exposure to PM.

Our study is the first to analyze the correlation between the asthma visits of children’s hospitals affiliated with Zhejiang University Medical College and the PM concentration in Hangzhou in the past 5 years. It was found that PM concentration in the atmosphere was positively correlated to the emergency department visits of children with asthma. In a further study in mice, we verified that short-term exposure to PM could aggravate allergic airway inflammation, in which IL-33 might be a promising target.

## RESULTS

### PM concentration and pediatric asthma visits have correlating seasonal fluctuations

**Figure 1:**
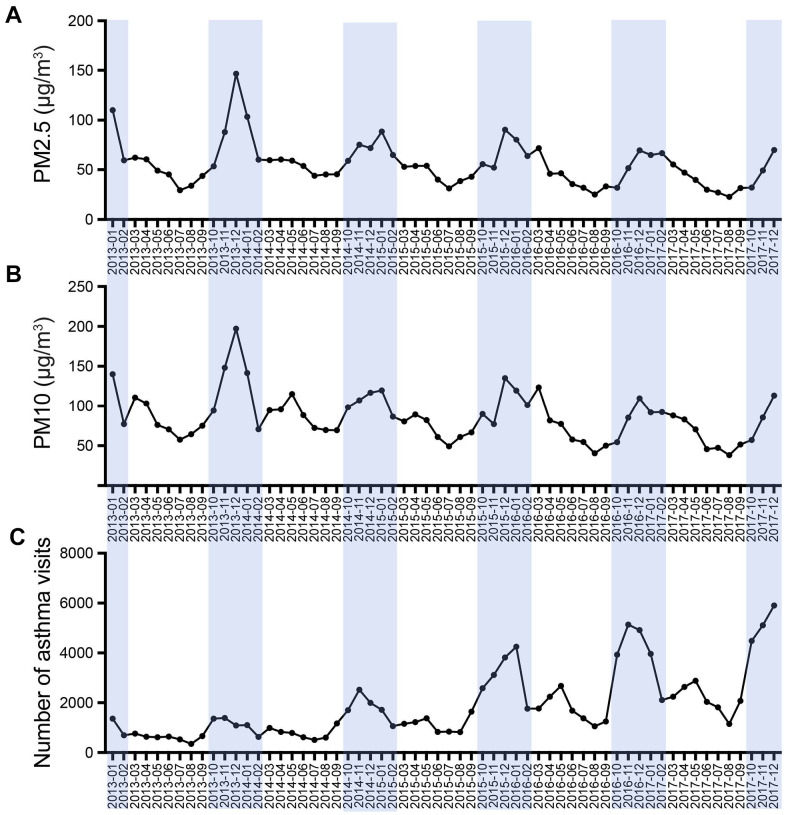
Temporal pattern of monthly average PM concentration (**A**, **B**) and asthma visits (**C**) during January 2013 to December 2017.

According to the data from the national air pollution prevention and control monitoring station, we described the monthly average PM concentration, including PM2.5 and PM10. It was found that the monthly average PM concentration in Hangzhou shows a strong correlation to seasons. Fine particulate pollution, including PM2.5 and PM10, aggravated in winter and moderated in summer. From January 2013 to December 2017, the monthly average PM2.5 concentration fluctuated between 22.8 and 146.5 μg/m^3^, while PM10 varies between 38.2 and 197.2 μg/m^3^ (Figure 1A, 1B).

In the 5-year study period, there were 16,779,739 visits involving children aged 0–14 in the outpatient and emergency department in the Children’s Hospital of Zhejiang University. We counted the number of children with asthma and found that the visits showed clear seasonal fluctuations (Figure 1C). From January 2013 to December 2017, the attendance of children with asthma (median) was 1377.50 per month, and in outpatient and emergency departments, the monthly ratio (median) of children with asthma in the total patients was 0.55% (Table 1).

**Table 1:** Average monthly outpatient and emergency visits in 5 years.

**Variables**	**<6y**	**≥6y**	**All ages**
Outpatient visits	161,227.00 (138,575.25-190,498.50)	56,584.50 (47,212.75-67,357.00)	219,485.00 (185,633.75-258,372.50)
Outpatient visits for asthma	1,005.00 (586.00-1,919.75)	313.50 (232.50-457.00)	1,360.50 (819.75-2,342.50)
Emergency visits	40,221.50 (32,030.00-53,310.00)	9,854.50 (8,763.00-13,654.50)	50,890.50 (40,371.25-66,558.75)
Emergency visits for asthma	5.00 (3.00-9.00)	5.50 (3.00-9.00)	9.50 (6.25-15.75)
Asthma visits	1,017.00 (592.00-1,955.25)	317.00 (239.00-464.75)	1,377.50 (827.50-2,453.25)
Total outpatient and emergency visits	202,946.00 (169,656.00-242,557.50)	68,469.00 (56,088.50-81,791.00)	266,244.00 (228,224.25-326,361.25)
Ratio	0.56% (0.37%-0.86%)	0.47% (0.34%-0.68%)	0.55% (0.37%-0.80%)

Expressed as Median (quartile).

Table 1: The table shows the monthly average visits of patients (medians and interquartile) in the Children’s Hospital of Zhejiang University during 2013–2017.

Note: Ratio, the proportion of asthmatic patients in the outpatient and emergency visits.

### The visits of asthma were associated with of PM2.5 and PM10 concentrations

**Figure 2:**
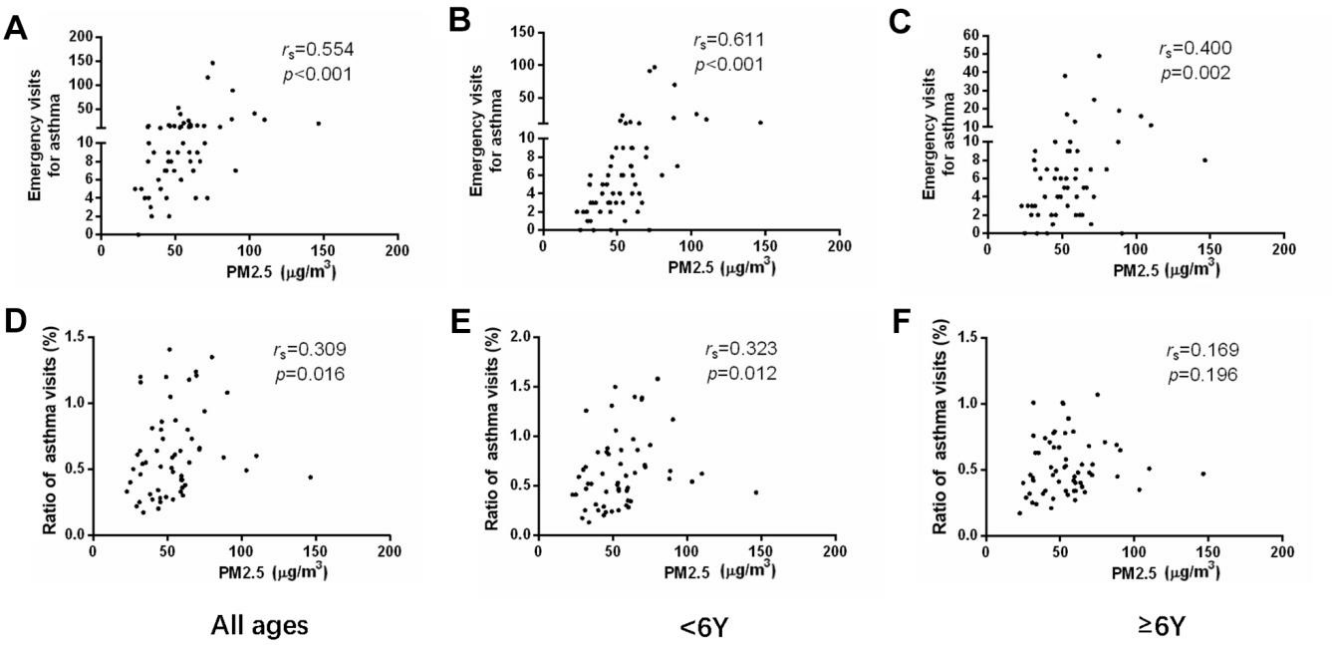
The correlations between PM2.5 concentration and emergency department visits (**A**–**C**) or ratio of asthmatic children to total patients in outpatient and emergency departments (**D**–**F**) in different age groups.

**Figure 3:**
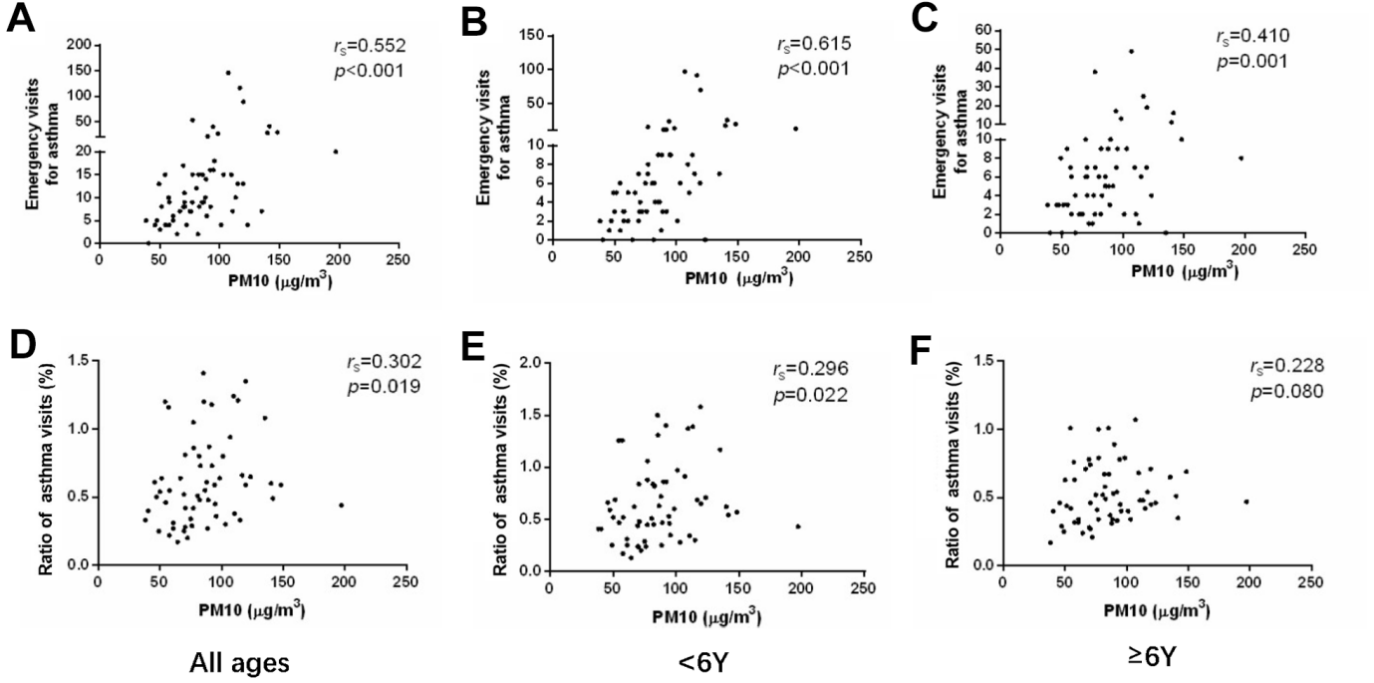
The correlations between PM10 concentration and emergency department visits (**A**–**C**) or ratio of asthmatic children to total patients in outpatient and emergency departments (**D**–**F**) in different age groups.

We analyzed the monthly visits of asthmatic children and the concentration of PM in the atmosphere. The concentrations of both PM2.5 and PM10 showed moderately positive correlation to emergency visits of asthma (r = 0.554, p < 0.001 and r = 0.552, p < 0.001) (Figures 2A, 3A and Tables 2, 3). Meanwhile, the concentrations of PM2.5 and PM10 were correlated to the ratio of asthmatic patients in total outpatient and emergency department visits (r = 0.309, p = 0.016 and r = 0.302, p = 0.019) (Figures 2D, 3D and Tables 2, 3). After grouping the children according to age, the emergency visits, and the ratio of asthmatic children in total patients under 6 years old were moderately correlated with the concentrations of PM2.5 (r = 0.611, p < 0.001 and r = 0.323, p = 0.012) (Figure 2B, 2E and Table 2) and PM10 (r = 0.615, p < 0.001 and r = 0.296, p = 0.022) (Figure 3B, 3E and Table 3), respectively. However, in the age group of 6 years and older, PM2.5 and PM10 concentrations generally correlated to the emergency visits (r = 0.400, p = 0.002 and r = 0.410, p = 0.001) (Figures 2C, 3C and Tables 2, 3), and there was no correlation between PM and the ratio of asthmatic visits in total outpatient and emergency department visits (r = 0.169, p = 0.196 and r = 0.228, p = 0.080) (Figures 2F, 3F and Tables 2, 3).

**Table 2:** The correlation between visits and PM2.5 concentration.

**Variables**	**<6y**		**≥6y**		**All ages**	
	**r_s_**	**p**	**r_s_**	**p**	**r_s_**	**p**
Outpatient visits	0.096	0.463	-0.450	<0.001	0.227	0.081
Outpatient visits for asthma	0.184	0.159	-0.060	0.649	0.154	0.240
Emergency visits	0.110	0.401	-0.003	0.983	0.089	0.497
Emergency visits for asthma	0.611	<0.001	0.400	0.002	0.554	<0.001
Asthma visits	0.192	0.143	-0.032	0.806	0.167	0.202
Total outpatient and emergency visits	0.106	0.419	-0.398	0.002	0.197	0.132
Ratio	0.323	0.012	0.169	0.196	0.309	0.016

Table 2: The table shows the correlation between visits and PM2.5 concentration in different age groups. 0.00 ≤ r_s_ < 0.30 means feeble correlation; 0.30 ≤ r_s_ <0.80 means moderate correlation; 0.80 ≤ r_s_ < 1.00 means strong correlation.

Note: Ratio, the proportion of asthmatic patients in the outpatient and emergency visits.

**Table 3:** The correlation between visits and PM10 concentration.

**Variables**	**<6y**		**≥6y**		**All ages**	
	**r_s_**	**p**	**r_s_**	**p**	**r_s_**	**p**
Outpatient visits	-0.041	0.758	-0.415	0.001	-0.179	0.170
Outpatient visits for asthma	0.193	0.139	0.005	0.967	0.168	0.199
Emergency visits	-0.054	0.680	0.065	0.620	-0.032	0.806
Emergency visits for asthma	0.615	<0.001	0.410	0.001	0.552	<0.001
Asthma visits	0.202	0.122	0.035	0.791	0.183	0.161
Total outpatient and emergency visits	-0.051	0.696	-0.357	0.005	-0.143	0.275
Ratio	0.296	0.022	0.228	0.080	0.302	0.019

Table 3: The table shows the correlation between visits and PM10 concentration in different age groups. 0.00 ≤ r_s_ < 0.30 means feeble correlation; 0.30 ≤ r_s_ < 0.80 means moderate correlation; 0.80 ≤ r_s_ < 1.00 means strong correlation.

Note: Ratio, the proportion of asthmatic patients in the outpatient and emergency visits.

### PM aggravated the HDM-induced airway inflammation

**Figure 4:**
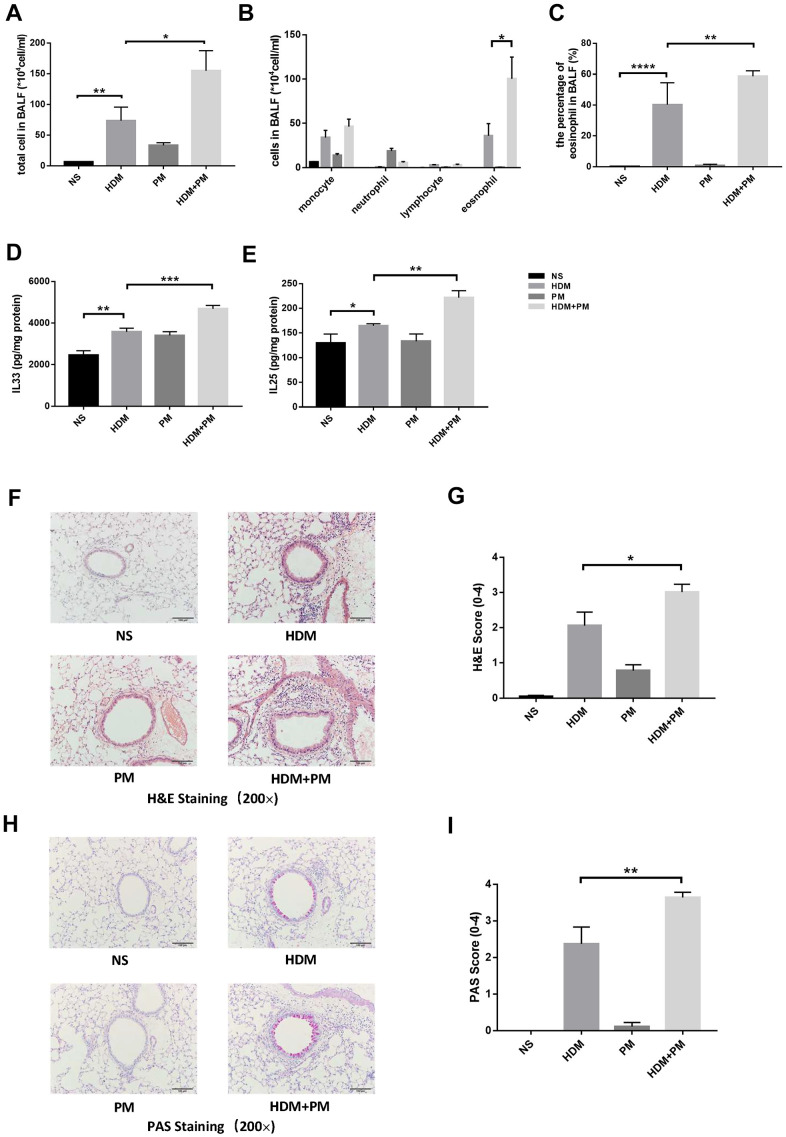
**PM2.5 aggravated the HDM-induced airway inflammation.** Male C57BL/6 mice (N = 8 for each group) were instilled intratracheally with NS, HDM, PM, or HDM+PM. Total inflammatory cells, cell differentiation of inflammatory cells, and the percentage of eosinophils in BALF were assessed (**A**–**C**). Protein levels of IL-33 (**D**) and IL-25 (**E**) in the lung tissues were measured using ELISA. Representative images of lung tissues stained with H&E (**F**) and PAS (**H**) were showed under the microscope (10 × 20 magnification). Inflammatory score (**G**) and mucus production (**I**) with semi-quantification (score: 0–4) was analyzed (N = 10 images for each group). Data were presented as Means ± SEM (*, P < 0.05; **, P < 0.01; ***, P < 0.001; ****P < 0.0001).

We established an HDM-induced allergic airway inflammation model with short-term PM exposure. Compared to the HDM group, PM intervention led to a significantly increasing tendency in total cell number and eosinophil (absolute and differential counts) in bronchoalveolar lavage fluid (BALF) (p < 0.05, p < 0.05, p < 0.01) (Figure 4A–4C). Additionally, lungs were homogenized to measure the concentration of cytokines by enzyme-linked immunosorbent assay (ELISA). The result showed that PM exposure resulted in the pulmonary accumulation of IL-33 and IL-25 in the HDM-induced airway inflammation (p < 0.001, p < 0.01) (Figure 4D, 4E). Hematoxylin and eosin (H&E) staining and Periodic Acid-Schiff (PAS) staining of pathological sections showed that infiltration of inflammatory cells around the airway and secretion of mucus in the airway in the HDM-induced airway inflammation were increased remarkably with PM intervention (p < 0.05, p < 0.01) (Figure 4F–4I).

### IL-33 neutralizing antibody exerted a protective role on PM-aggravated HDM-induced airway inflammation

**Figure 5:**
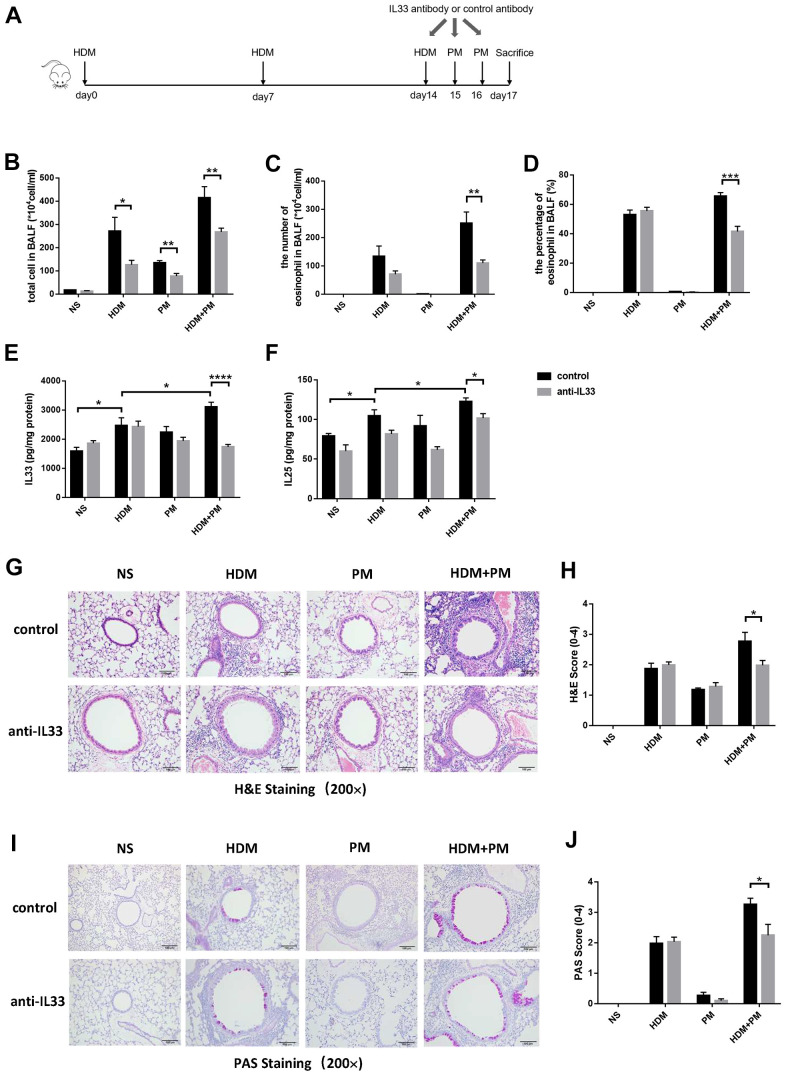
**IL-33 antibody alleviated the aggravation of airway inflammation caused by PM exposure in the HDM-induced allergic mice.** Male mice in the control and experimental groups were injected intraperitoneally with IL-33 antibody or control antibody, respectively (N = 8 for each group). Protocol for the IL-33 antibody or control antibody intervention (**A**). Total inflammatory cells, eosinophil proportion, and eosinophil number were analyzed (**B**–**D**). Protein levels of IL-33 (**E**) and IL-25 (**F**) in the lung tissues were measured using ELISA. Representative images of lung tissues stained with H&E (**G**) and PAS (**I**) were showed under the microscope (10 × 20 magnification). Inflammatory score (**H**) and mucus production (**J**) with semi-quantification (score: 0–4) were analyzed under the microscope (10 × 20 magnification). Data were presented as means ± SEM (*, P < 0.05; **, P < 0.01; ***, P < 0.001; ****, P < 0.0001).

To further investigate the effect of IL-33 on the PM exposure-aggravated airway inflammation, we administered an IL-33 neutralizing antibody before PM exposure in HDM-induced asthma models (Figure 5A). We evaluated the number of total cells, and cell differential counts in the BALF. Compared to the control group, the allergic mice exposed to PM had a prominent decrease in total cell number and eosinophil (absolute and differential counts) in BALF with the administration of IL-33 antibody (p < 0.01, p < 0.01, p < 0.001) (Figure 5B–5D). The protein level of IL-33 and IL-25 in the lung tissues of asthmatic mice were increased markedly after PM intervention (p < 0.05, p < 0.05) and decreased dramatically with the IL-33 antibody administration (p < 0.0001, p < 0.05) (Figure 5E, 5F). Moreover, histological analyses revealed the infiltration of inflammatory cells and the secretion of mucus of the PM-exposed asthmatic mice with IL-33 antibody were significantly alleviated versus the control group (p < 0.05, p < 0.05) (Figure 5G–5J).

## DISCUSSION

PM is one of the most harmful constituents of air pollution to human health. Exposure to PM, especially PM2.5, can increase hospitalizations and mortality of respiratory diseases, including asthma, impaired lung function, and even lung cancer [16, 17]. The epidemiological investigation found that the emergency department visits of asthmatic patients increased along with the higher levels of PM2.5 concentration, and children were more susceptible to increased PM2.5 than adults [18]. In addition, the severity of the symptoms of childhood asthma and the incidence of severe asthma is believed to be related to the concentration of PM2.5 [19]. Since the compositions of PM and the incidence of asthma were different between regions, we analyzed the correlation between the visits of asthmatic children and PM concentration in the Hangzhou area. We verified that PM 2.5 and PM10 concentrations positively correlated to both the number of asthmatic children in the emergency department and the ratio of asthmatic patients in total visits of outpatient and emergency departments.

Several studies have shown that different populations respond differently to atmospheric PM. In children and people over 65 years old, PM2.5 has a greater impact on the respiratory system than other populations [20]. Therefore, we further divided the asthmatic children into two groups (children < 6 years old and ≥ 6 years old). The results confirmed that in children younger than 6 years old, the correlation between PM concentration and asthma aggravation was more obvious than the older children. Consequently, young children with asthma are more sensitive to the influence of PM, which might be due to the incomplete development of the children’s respiratory and immune systems. This also suggests that more attention is needed to the impact of PM on children, especially young children when formulating policies on air pollution.

It has been reported that long-term repeated exposure to PM can lead to increased airway inflammation in the ovalbumin (OVA)-induced asthma model [21, 22]. However, there are few studies on the short-term exposure to PM in the HDM-induced airway inflammation model. We established the HDM-induced airway inflammation model and substantiated that short-term PM intervention aggravated the airway inflammatory response in mice. Histological analysis further confirmed that PM exacerbated the infiltration of inflammatory cells around the airway and secretion of mucus in the airway. These results were essentially consistent with the effect of long-term PM exposure on airway inflammation in the asthma model [21, 22]. This indicates that short-term intervention of PM induced the increase of eosinophils in the airway and aggravated the airway inflammation of asthma.

Previous studies have suggested that the activation of inflammatory-associated cells might be involved in the molecular mechanism of PM-induced airway inflammation [23, 24]. *In vitro*, PM was associated with increased release of cytokines, such as IL-33, IL-1, IL-6, and IL-8 [25–27]. But *in vivo*, the relationship between PM and the release of associated cytokines remains uncertain [28]. Our study revealed that the airway inflammatory response aggravated by PM intervention was accompanied by elevated expression of IL-33. IL-33 is expressed in the epithelial cells of the lungs and plays an important role in type-2 innate immunity by activating eosinophils, which are associated with allergic inflammation [29, 30]. Antibodies to IL-33 are evaluated in ongoing asthma-related clinical studies [31]. However, few studies have explored the role of IL-33 in HDM-induced asthma models under PM exposure. We confirmed that IL-33 neutralizing antibody could alleviate the aggravated airway inflammation induced by PM exposure in asthmatic mice. This verifies that the IL-33 antibody exerted a protective role on PM-aggravated HDM-induced airway inflammation. Nevertheless, there is no obvious alleviation of airway inflammation in the HDM-induced asthma mice treated with IL-33 antibody, which might because the airway inflammation induced by HDM had basically formed when the IL-33 antibody was administrated.

As stated, our study findings suggest that there is a positive correlation between the concentration of PM and the pediatric asthma visits, and the increase in risk was strongest in emergency visits, especially in children under 6 years old. Additionally, short-term exposure to PM aggravated the allergic airway inflammation, in which IL-33 might play an important role. Thus, these data provide insights into novel preventive and/or protective approaches for PM-induced airway disorders.

## MATERIALS AND METHODS

### City-level estimates of environmental variables

In this research, we analyze the meteorological data in Hangzhou. The monthly average PM2.5 and PM10 concentrations from January 2013 to December 2017 were derived from 11 National Air Pollution Monitoring Stations located in Hangzhou. These monitoring points are distributed throughout the city, including urban and suburban areas, and can objectively and accurately reflect the concentration of atmospheric particles in this region.

### Health and population outcome data

The study subjects were children suffering from asthma. Data on outpatient and emergency visits of asthma patients were obtained from the Children’s Hospital of Zhejiang University. The children’s hospital of Zhejiang University is the largest children’s hospital in Zhejiang province, with monthly outpatient and emergency visits reaching 20,000 or more. We collected the average monthly outpatient and emergency visits of asthmatic patients in children’s hospital and compared them with the total hospital visits. This data can reflect the incidence of asthma in the total patient population and asthma attacks. The study was approved by the ethics committee of the Children’s Hospital, Zhejiang University School of Medicine.

### Animals

C57BL/6 mice (wild-type, aged 6–8 weeks) were purchased from the Slac Laboratory Animal Co. Ltd. (Shanghai, China). All mice were housed in the Laboratory Animal Center of Zhejiang University, Hangzhou, China. All experiments involving animals were maintained in a specific-pathogen-free facility and were given a standard diet with normal drinking water. The room temperature was precisely maintained at 23 ± 2° C, and the humidity was kept at 50% ± 10%. The study was in accordance with the stipulations and protocols approved by Ethics Committee for Animal Studies at Zhejiang University.

### HDM-induced allergic airway inflammation in the mouse model

Freeze-dried HDM extract was purchased from Greer’s laboratory (10.52 EU/mg endotoxin) and dissolved in normal saline to a concentration of 2 μg/μL. The HDM-induced asthma model was established in mice at 6 weeks old. Mice in the HDM group were anesthetized and challenged with 50 μL of HDM (100 μg per mouse) via intratracheal instillation on days 0, 7, and 14. The control mice in the NS group were given NS. Mice were euthanized using a sodium pentobarbitone overdose intraperitoneally (i.p) at 72 h after the final allergen challenge on day 17.

### PM intervention

PM was purchased from the National Institute of Standards and Technology, and the main components of PM were PAHs, nitro-substituted PAHs, polychlorinated biphenyl congeners, chlorinated pesticides, and inorganic constituents. Allergic airway inflammation was established in the HDM+PM group as previously described. After the last HDM administration on day 14, mice were given 50 μL of PM (100 μg per mouse) per day by intratracheal instillation on days 15 and 16. The control mice in the PM group were administered 50 μL of NS on days 0, 7, and 14, and PM (100 μg per mouse) on days 15 and 16 via intratracheal instillation. Mice were euthanized using a sodium pentobarbitone overdose intraperitoneally, and parameters were analyzed on day 17.

### IL-33 neutralizing antibody intervention

Mice were divided into IL-33 antibody and control groups. In each group, NS, HDM, PM, and HDM+PM were treated according to the protocol described above, respectively. Anti-mouse IL-33 and the isotype control antibodies (R&D Systems) were resuspended in sterile PBS and administered three consecutive times from day 14 to 16 with 2 μg/mouse per day via intraperitoneal injection (i.p).

### BALF collection and differential cell count

A cannula was inserted in the trachea, and then the left lungs were administered with 0.4 mL PBS three times for 1 mL BALF. Cells in BALF were counted under a microscope with a double-blind method. After cell count, BALF was centrifuged, spun onto glass slides, and stained with Wright-Giemsa. The completed slides were placed under a microscope to count the number of differential cells, and at least 200 cells were observed.

### Histologic analysis

The left lung tissues were fixed with formaldehyde for 24 h and then embedded in paraffin. The sections were stained with H&E and PAS. H&E staining sections were assessed for the airway inflammatory situation and quantitated as previously described [32]. PAS staining sections were assessed for mucus production of goblet cells according to the previous studies [33]. All slides were examined in a randomly blinded fashion by two independent investigators.

### Enzyme-linked immunosorbent assay (ELISA)

The lungs of each group were cut into small pieces, homogenized with RIPA lysis buffer, and centrifuged at 12,000 g for 15 min. Then, the supernatant was collected. IL-33 (Biolegend, CA, USA) and IL-25 (MultiScience, Germany) expression were quantified by ELISA kit according to the manufacturer’s protocol.

### Statistical analyses

Statistical Package for the Social Sciences (SPSS) version 23.0 (SPSS, Chicago, IL, USA) was used for the clinical data analysis. PM2.5 and patient numbers as nonparametrically distributed data were expressed as medians and interquartile range. Accordingly, correlations among the selected variables were examined by Spearman rank correlation analysis.

Statistical analyses were performed with one-way ANOVA for the comparison of different groups in animal studies. Data were expressed as the means ± SEM. The analyses and graphs were performed with GraphPad Prism 7.0 software (GraphPad Software Inc., CA, USA). P-values < 0.05 were considered statistically significant.
